# Multi‐stage automatic and rapid ablation and needle trajectory planning method for CT‐guided percutaneous liver tumor ablation

**DOI:** 10.1002/mp.17450

**Published:** 2024-10-10

**Authors:** Shengwei Li, Fanyu Zhou, Yumeng Zhang, Sheng Xu, Yufeng Wang, Lin Cheng, Zhixin Bie, Bin Li, Xiao‐Guang Li

**Affiliations:** ^1^ Minimally Invasive Tumor Therapy Center, Beijing Hospital, National Center of Gerontology Institute of Geriatric Medicine Chinese Academy of Medical Sciences Beijing China; ^2^ Graduate School Peking Union Medical College Beijing China; ^3^ Research and Development Center Hygea Medical Technology Co., Ltd. Beijing China

**Keywords:** computer‐assisted ablation planning, liver tumor, needle trajectory planning

## Abstract

**Background:**

Computer‐assisted planning methods have increasingly contributed to preoperative ablation planning; however, these methods cannot automatically obtain the final optimal solution within a short time and are rarely validated in practice, greatly limiting their clinical applicability.

**Purpose:**

We aimed to propose a full‐automatic multi‐stage ablation and needle trajectory planning method for CT‐guided percutaneous liver ablation to attain the final optimal plans under multiple clinical constraints rapidly.

**Methods:**

Our proposed method integrates the ablation zone planning fulfilling complete tumor coverage and critical structure avoidance while reaching a trade‐off between ablation number and healthy tissue damage, and needle trajectory planning under multiple clinical constraints. Our needle trajectory planning determines feasible skin entry regions based on hard constraints, where the multi‐objective optimization (MOO) considering soft constraints is performed using the Pareto Optimality and Technique for Order of Preference by Similarity to Ideal Solution (TOPSIS) methods for the final optimal solution. The performance of our proposed method was evaluated on 30 tumors of various characteristics from 23 patients and clinically validated in five clinical cases.

**Results:**

Our proposed method achieved 99.8% treatment zone coverage and 40.5% ablation efficiency without involving critical structures, and completely satisfied multiple clinical constraints in all needle trajectory planning results. The average planning time was 23.6 s for tumors of different sizes. All the plans were considered clinically acceptable by the doctors’ evaluation. Our method achieved complete tumor coverage without complications in clinical case validation.

**Conclusion:**

Our proposed planning method can generate a final optimal plan satisfying multiple clinical constraints within a short time, potentially facilitating preoperative planning for hepatic tumor ablation.

## 1 INTRODUCTION

Image‐guided percutaneous ablation is a minimally invasive therapy and become a clinically favored alternative treatment for liver tumors due to fewer complications, rapid rehabilitation, and low costs.^1^, ^2^, ^3^, ^4^ During ablation procedures, an ablation needle is percutaneously inserted into the tumor under imaging guidance, and cancerous cells are destroyed in situ.^5^ Successful ablation should achieve complete ablation of the tumors, minimal damage to healthy tissues, and avoidance of critical structures such as vital organs and large blood vessels.^6^ Unreasonable needle insertion paths may cause incomplete ablation or higher complication risks, affecting the effectiveness and safety of ablations.^7^, ^8^ Therefore, therapeutic success crucially relies on preoperative ablation needle planning.

Ablation and needle trajectory planning mainly depends on the physician's experience with the guidance of computed tomography (CT) or magnetic resonance imaging (MRI).^9^ However, intuitive patient‐specific information cannot be comprehensively presented in a 2D slice‐based manner, possibly impairing surgical decision‐making.^10^ Although doctors can obtain more visualized spatial relationships of the tumor and its surrounding structures from 3D reconstructed images,^11^ manual planning remains time‐consuming and challenging due to complex anatomy and various clinical constraints. Manual surgical planning for large tumors is confronted with higher challenges: time‐consuming schemes, demanding surgical experiences, complicated configurations of multiple ablation zones, and interactions between needle trajectories.^12^, ^13^, ^14^ Several automatic planning methods have recently been proposed to solve the above problems.

Automatic ablation needle planning involves ablation planning and needle trajectory planning. In the ablation planning, the expected treatment zones include the tumor with an enlarged 5–10 mm safety margin to completely eradicate the cancerous and infiltrative tissues against local recurrence.^15^ The simulated ablation zones should completely cover the treatment zones without involving critical structures.^16^, ^17^ For large tumors, multiple ablation zones are geometrically configured and optimized to minimize the number of ablation needles and damage to healthy tissues.^18^ In needle trajectory planning, needle trajectory is typically abstracted as the line between a target point (tumor) and a skin entry point, and all the trajectories are evaluated according to various clinical constraints to determine the optimal solution.^19^ Particularly, the feasible skin entry regions are initially restricted based on hard constraints such as no collisions with surrounding obstacles, and a filtered set of candidate trajectories is optimized using soft constraints that are representative clinical parameters, such as the distance to critical structures.^20^ Under multiple constraints from clinical experience, needle trajectory planning can be mathematically abstracted into multi‐objective optimization (MOO) problems,^18^, ^21^ which generally do not have a single optimal solution due to the contradiction among multiple objective functions and their priority.

Prior computer‐assisted methods have contributed to the solutions to MOO of automatic ablation needle planning. In earlier studies, Baegert et al.^22^ automatically performed MOO for single‐needle trajectory planning using the downhill simplex method. Schumann et al.^23^ introduced a weighted combination of cylindrical projections for single‐needle trajectory MOO to generate a list of potential paths (“constraint map”). To avoid weight adjustments, Seitel et al.^24^ first introduced Pareto Optimality into MOO problems in needle trajectory planning. Pareto optimal solutions are non‐dominated solutions that systematically take into account all the optimized objectives, which are compatibly applied to MOO problems. Then, Schumann et al.^25^ were inspired and used Pareto Optimality integrated simulation approximation in single‐needle MOO. However, these single‐needle planning methods did not cover the multi‐needle optimization for large tumors. For the multi‐needle planning, Chen et al.^26^ proposed a clustering algorithm that semi‐automatically computed the targets and corresponding conical puncture regions based on tumor geometric simulation. Liang et al.^27^ combined the set cover‐based model and Pareto optimization for the mathematically optimal MOO solution, but probably resulting in sub‐optimal solutions due to the limit of optimization time. Li et al.^28^ proposed a multi‐constraint planning method for trajectory optimization of large tumor ablations by setting adjustable safety margins, however, multiple solutions were sometimes generated due to the determination of the needle number. Li et al.^29^ simplified 3D ablation planning into 2D insertion path and ablation position planning using orthogonal projection, obtaining a clinically acceptable plan. Li et al.^30^, ^31^ developed multi‐needle planning methods using Pareto optimization for tumors with different sizes and obtained a series of Pareto optimal solutions, which required further determination by the physician. However, the optimized results from these approaches above were clinically feasible needle paths or a set of Pareto optimal solutions, but not the global optimal solution, and it still decisively relies on physician experience when determining the final planned trajectory. To pursue a mathematically optimal MOO solution within a short time, the Technique for Order of Preference by Similarity to Ideal Solution (TOPSIS) method, a multi‐criteria decision analysis (MCDA) algorithm,^32^ was incorporated into our optimization to rank the Pareto optimal solutions quantitatively, which can lessen dependence on doctor's surgical experience and hesitations on the final optimal solution.

Therefore, we aimed to propose a multi‐stage ablation and needle trajectory planning method for CT‐guided percutaneous liver tumor ablation that integrates ablation zone and needle trajectory planning under multiple clinical constraints, to automatically and rapidly attain the final optimal plan, and evaluate its performance on retrospective CT data and clinical cases.

## 2 METHODS

### 2.1 Dataset

This retrospective study received ethical approval from the Institutional Review Committee of our institution (2022‐BJYYEC‐361‐01) and the informed consent was waived.

**FIGURE 1:**
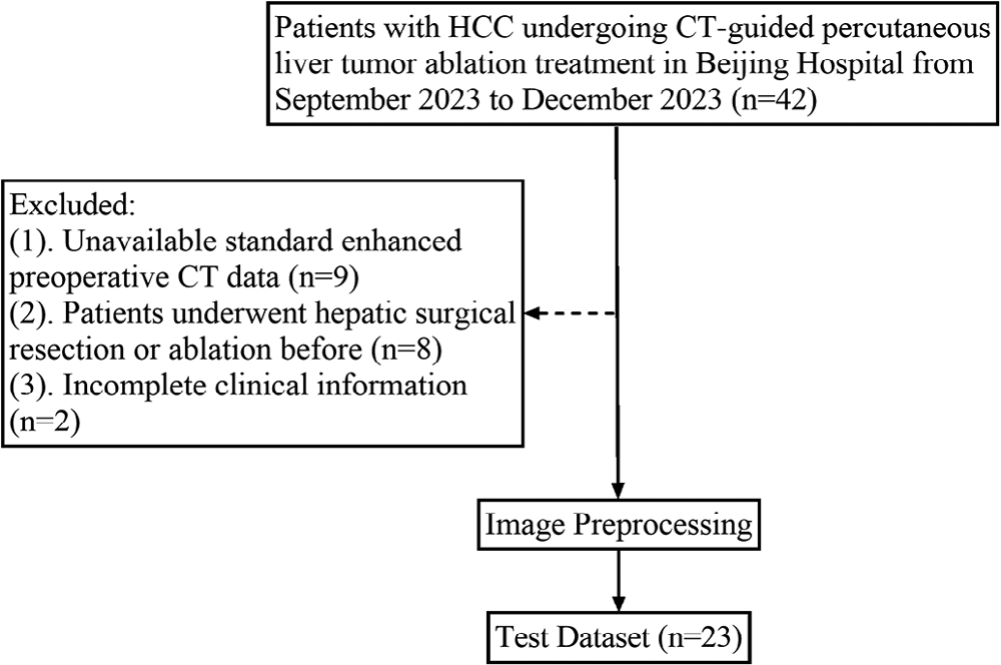
The patient selection workflow.

A total of 42 patients with histologically confirmed hepatocellular carcinoma (HCC) undergoing CT‐guided percutaneous liver tumor ablation treatment in our institution from September 2023 to December 2023 were screened initially and 23 patients with 30 tumors were eventually enrolled in the Test dataset based on the inclusion and exclusion criteria (Figure 1). The inclusion criteria were as follows: (a) patients with histologically confirmed HCC, and with Barcelona Clinic Liver Cancer (BCLC) 0‐A or China Liver Cancer Staging (CNLC) Ia‐IIa; (b) no treatment before ablation; (c) complete preoperative contrast‐enhanced CT data and clinical information. The exclusion criteria were as follows: (a) unavailable imaging data; (b) history of hepatic surgical resection, transplantation, or ablation treatment; (c) incomplete clinical information. Multiphase contrast‐enhanced CT data were acquired by a multi‐slice CT scanner (GE, GE Health care, Boston, USA). All CT data were presented with a slice thickness of 1.25 mm for image preprocessing. Clinical information, including age, gender, number of tumor lesions, BCLC and CNLC stage, and tumor size, was obtained from the medical records.

## 3 NEEDLE TRAJECTORY PLANNING METHOD

**FIGURE 2:**
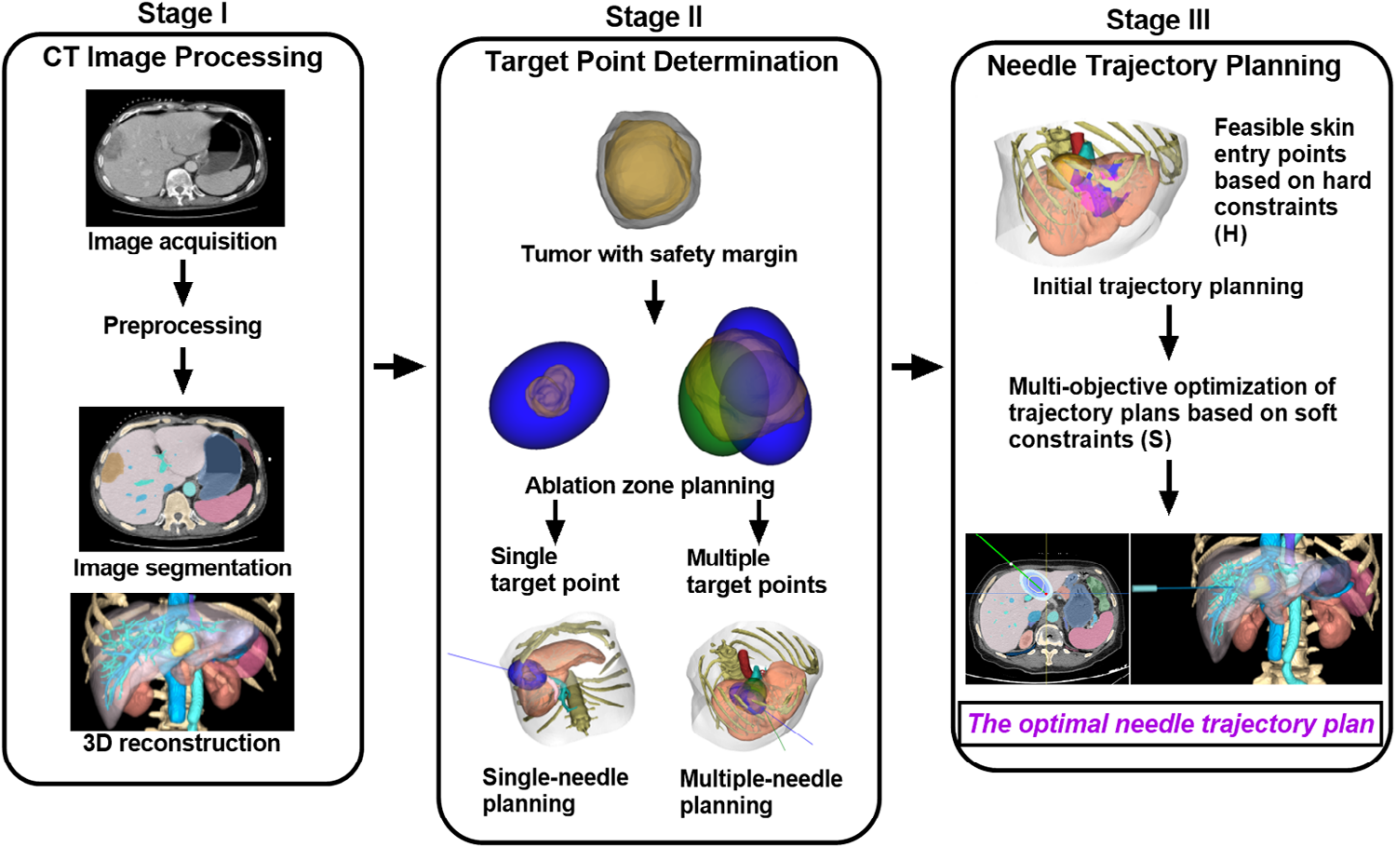
The workflow chart of the proposed multi‐stage ablation and needle trajectory planning method.

As illustrated in Figure 2, our planning method involves three stages. First, CT scans are preprocessed for segmentation and 3D reconstruction of the tumor and anatomical structures nearby. Then, the number and location of the target point are optimized and determined by multi‐constrained ablation zone planning. Finally, feasible skin entry regions are determined based on hard constraints, followed by two‐stage MOO based on soft constraints for the optimal needle trajectory plan.

### 3.1 Image preprocessing

After image acquisition, CT data are all resampled to 1×1×1 mm voxel size for computational simplicity. Then, tumors and anatomical structures, including skin, abdominal organs, and blood vessels are manually segmented and annotated by a senior radiologist (Dr S.L. with 8 years of experience in abdominal radiology) using ITK‐SNAP 3.6 software.^33^ The initial segmentation masks are critically checked and refined by an expert radiologist (Dr B.L., with 20 years of experience in abdominal radiology). Finally, 3D reconstructions are accomplished based on the final segmentation results.

### 3.2 Target point determination

#### 3.2.1 Treatment zone volume

**FIGURE 3:**
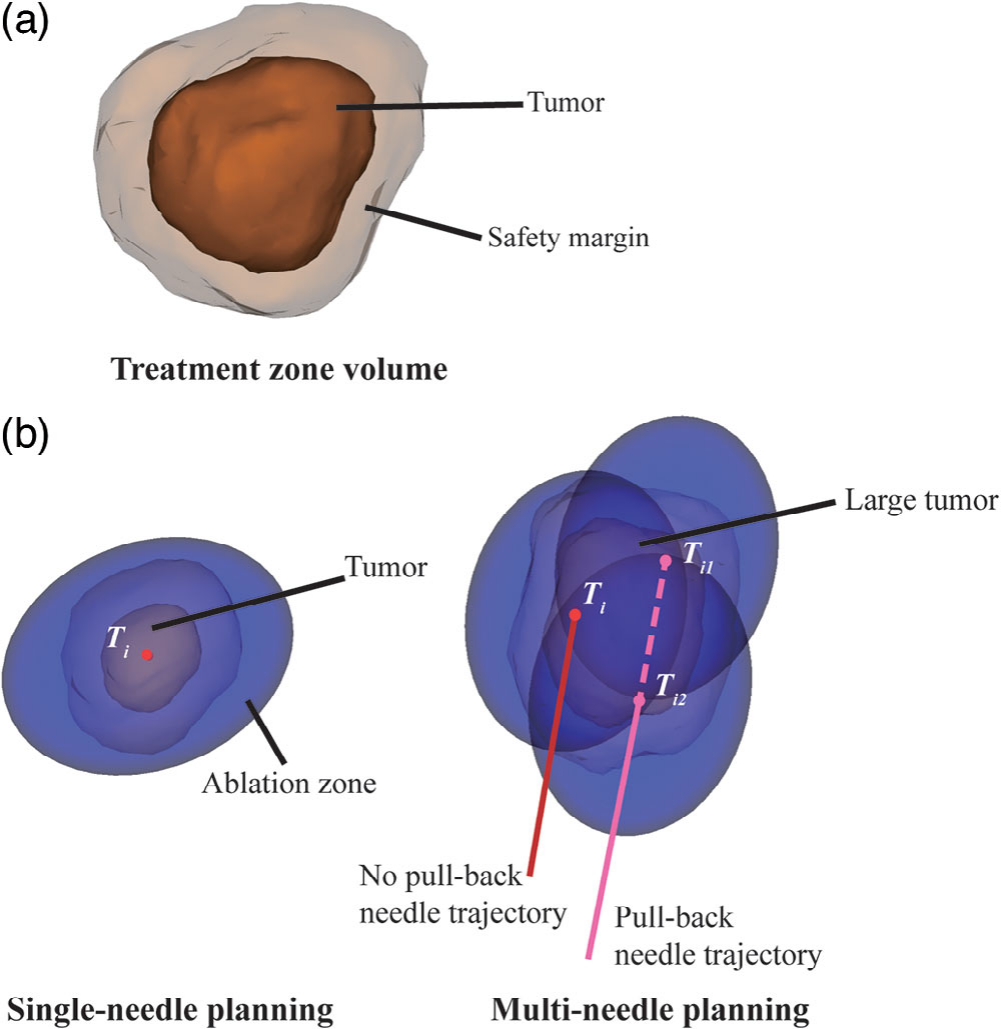
(a) The treatment zone volume, including the tumor volume with a safety margin. (b) The target point determination in single‐needle and multi‐needle ablation planning. *T_i_* represents the target point of the needle trajectory path *p_i_* without pull‐back; $T_{i1}$ and $T_{i2}$ are the first‐push, and second‐pull target points in the pull‐back needle trajectory, respectively. The pull‐back trajectory involves multiple pull‐back target points $T_{i1},T_{i2},\text{…},T_{\mathrm{ik}}$, where 𝑘 is the number of pull‐back target points.

The number and location of ablation zones should be formulated and optimized to adequately cover the tumor and its safety margin without overlapping the critical structures while minimizing damage to healthy tissues. To prevent the local recurrence after ablation, a 5 mm safety margin is added around the extracted tumor volume (TV) using morphological dilation, and the volume of the tumor with a 5 mm safety margin is defined as the treatment zone volume (TZV) (Figure 3a).

#### 3.2.2 Ablation zone planning

In our method, the ablation zone is simulated as a geometric ellipsoid with specific parameters depending on the ablation needle type, time, and power, and each ablation zone is assumed to perform independently and simultaneously without mutual interference. Since the centroid of each ablation ellipsoid is considered the target point of each needle trajectory in single‐ or multi‐needle planning, single or multiple target points are determined by the location or configuration of the ablation zone, respectively (Figure 3b). For higher flexibility and adaptability to various clinical situations, our approach technically allows for simulated ablation zones with adjustable sizes defined by physicians.

Given the fixed ablation zone, the required ablation number should be minimized to lessen the damage to healthy tissues while fulfilling complete tumor coverage. Taking both ablation efficiency and clinical constraints into account, the spatial configuration of each ablation zone is mathematically optimized. Particularly, simulated multi‐ablation zones should keep a proper distance from each other, avoiding lower efficiency caused by too many overlapped zones. Considering the safety and heat‐sink effect, critical structures including the heart, lungs, stomach, spleen, kidneys, gallbladder, pancreas, bile ducts, intestinal tracts, bones, and large blood vessels (diameter > 3 mm) cannot be involved by the simulated ablation zones. Under the multi‐constrained optimization, the centroid of each ablation ellipsoid, as well as the target point of each trajectory, is determined.

Clinically, the pull‐back technique refers to the sequential implementation of multiple ablations along one trajectory path in one needle puncture, where the needle tip is first inserted into the deepest position and pulled back along a trajectory (Figure 3b). This technique is widely applied to RFA and WMA treatments for large tumors to lessen the number of ablation needles and punctures, reducing complication risks and surgical costs.^21^, ^31^, ^34^ The pull‐back technique is systematically incorporated into our ablation planning for higher practicability.

#### 3.2.3 Ablation planning algorithm

##### Number of ablation needles

Assuming the ablation zone of each needle can cover the TZV at a ratio η (typically 1≤η≤5), the required number of ablation zones n can be estimated by the following formula:

$$n\approx \frac{V_{\mathrm{TZV}}}{\eta \times V_{\mathrm{probe}}}$$

Considering the practical layout and overlap of ablation zones, the final number should be rounded up and appropriately increased to ensure complete tumor coverage.

##### Target points for ablation needles

The ablation planning aims to establish an optimal strategy that ensures complete tumor coverage and critical structure avoidance while minimizing damage to healthy tissues.

- Ensuring complete tumor coverage: The ablation zone must cover the TZV entirely.
- Avoiding critical structures: The ablation zone should avoid overlapping critical organs or structures, such as large blood vessels.
- Minimizing damage to healthy tissue: The ablation zone should be configured to lessen the healthy liver tissue within the ablation volume.
- Ablation zone shape: The ablation zone is approximated as an ellipsoid centered at the ablation needle tip or a configuration of multiple ellipsoids.

p=(x,y,z) is defined as the 3D coordinates of the ablation needle tip.

θ,ϕ,ψ are defined as the rotation angles of the ablation needle (around the x, y, and z axes, respectively).

E(p,θ,ϕ,ψ) is defined as the rotated ablation ellipsoid centered at *p*.

The constrained optimization can be formulated as follows:

(1) Multiple constraints:
• Complete tumor coverage constraint: the configuration of multiple ablation ellipsoids must completely cover the tumor.
$$\begin{array}{ccc} G(E) & = & \mathrm{Volume}\left(T\right)\\ & & -\;\mathrm{Volume}\;\left(T\cap \overset{n}{\underset{i\;=1}{⋃}}E\left(p_{i},\theta_{i},\phi_{i},\psi_{i}\right)\right)=0 \end{array}$$
  where T represents tumor regions. This constraint ensures that the TZV is entirely covered by ablation zones.
• Avoiding critical structures:
$$\begin{array}{ccc} H\left(E\right) & = & \sum_{j=1}^{m}Volume\left(S_{j}\cap \overset{n}{\underset{i=1}{⋃}}E\left(p_{i},\theta_{i},\phi_{i},\psi_{i}\right)\right)=0,\\ & & j=1,2,\text{…},m \end{array}$$

The set of constraints ensures that the ablation zones do not involve critical structures $S_{j}$.

(2) Objective function:
• Minimizing damage to healthy tissue:
$$\begin{array}{ccc} & & R\;\left({\left\{p_{i},\theta_{i},\phi_{i},\psi_{i}\right\}}_{i\;=1}^{n}\right)\\ & & \;=\;\mathrm{Volume}\left(H\cap \overset{n}{\underset{i\;=1}{⋃}}E\left(p_{i},\theta_{i},\phi_{i},\psi_{i}\right)\right) \end{array}$$
  where H denotes the volume of healthy tissue, and $\cup_{i\;=1}^{n}E\left(p_{i},\theta_{i},\phi_{i},\psi_{i}\right)$ represents the ablation zones of multiple ellipsoids. This term aims to minimize the volume of healthy tissue *H* intersecting with the ablation zone ellipsoids $E(p_{i},\theta_{i},\phi_{i},\psi_{i})$.

Using the augmented Lagrangian method,^35^ the multi‐constrained optimization problem is transformed into an unconstrained optimization problem and solved by an iterative method. When planning the configuration of multiple ablation ellipsoids, the damage volume of each ellipsoid to healthy tissue, the joint tumor coverage rate of ellipsoids, and the corresponding penalty terms were comprehensively considered in the modified objective function, which allows for an unconstrained space in the optimization process while gradually approaching a solution satisfying the constraints.

The augmented Lagrangian function is as follows:


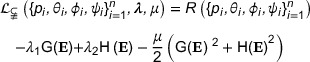

Where $\lambda =\{\lambda_{1},\;\lambda_{2}\}$ denotes the Lagrange multiplier and μ denotes the penalty parameter used to adjust the weight of penalty term. The iterative solution is stepped in Materials S1.

### 3.3 Needle trajectory planning

#### 3.3.1 Determination of feasible skin entry regions

**FIGURE 4:**
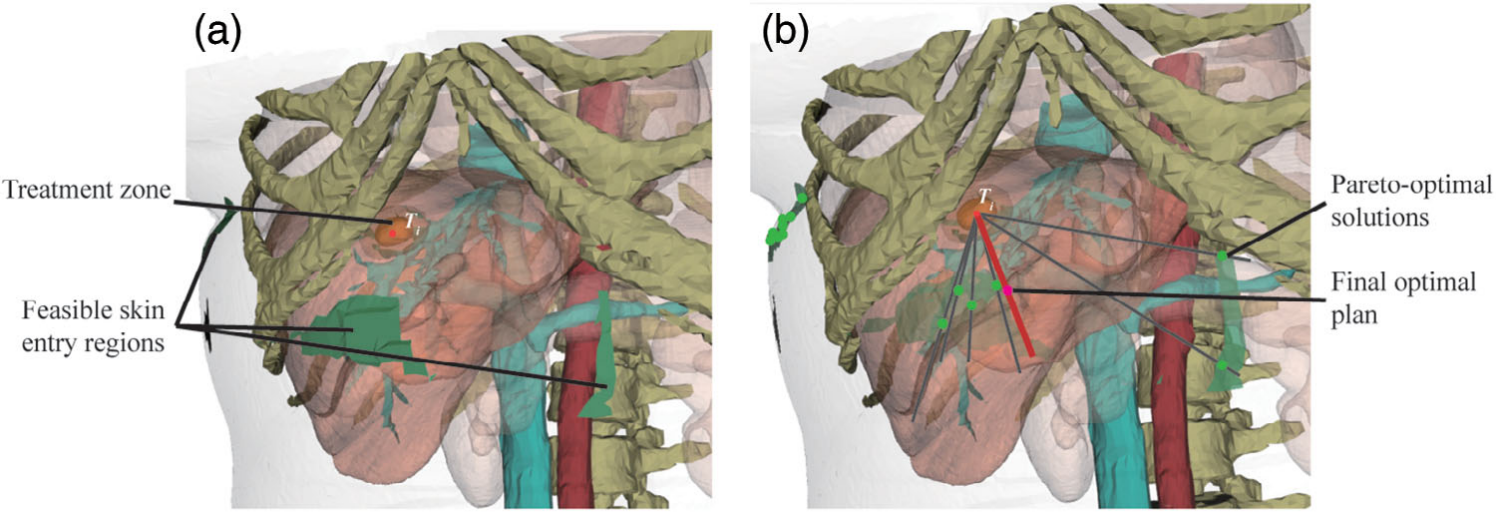
The procedures of needle trajectory planning. (a) Determination of feasible skin entry regions (green regions). (b) Multi‐objective optimization for Pareto‐optimal solutions (gray lines) and the final optimal plan (red line).

The feasible skin entry regions for each target point are determined based on six hard clinical constraints (Figure 4a), comprehensively considering the critical structure collision, needle length, insertion angles, transhepatic depth, puncture scope, and needle collision constraints (H1‐H6, listed in Table 1) in our initial trajectory planning. The feasible regions satisfying the hard constraints effectively restrict the searchable scope for skin entry points of the iterative candidate trajectory paths, improving computational speed and quality of candidate trajectory sets.

**TABLE 1:** Clinical constraints in the needle trajectory planning.

Hard constraints	Description	Objective Function	Notes
H1	Needle trajectories $p_{i}$ cannot pass through the critical structures including the heart, lungs, stomach, spleen, kidneys, gallbladder, bile ducts, intestinal tracts, bones, and large blood vessels (hepatic artery, hepatic vein, and portal vein).	$p_{i}\cap \;S_{citical}=\emptyset$	the set of critical structures $S_{citical}$
H2	The needle trajectory length $L_{i}$ must be less than the maximum length of the ablation needle $L_{max}$​.	$L_{i}<L_{max}$​	
H3	The insertion angle $\theta_{i}$ between the needle trajectory and the liver capsule must be greater than $20^{\circ }$ to prevent the ablation needle from gliding on the liver capsule.	$\theta_{i\;}>20^{\circ }$	
H4	The transhepatic trajectory depth $d_{i}$ must exceed 5 mm.	$d_{i}>5mm$	
H5	The horizontal deflection angle $\alpha_{i}$ of the needle trajectory must be restricted within the range of $30^{\circ }$ to $150^{\circ }$ above the operating table.	$30^{\circ }\leq \alpha_{i}\ll 150^{\circ }$	
H6	In multi‐needle ablation planning, the distance $d_{ij}$ between adjacent needle trajectories must exceed 5 mm for no inter‐trajectory collisions.	$d_{ij}>5mm$	the shortest distance $d_{ij}$ between adjacent needle trajectory $p_{i}$ and $p_{j}$

Soft constraints	Description	Objective function	Notes
S1	The vertical deflection angle $\beta_{i}$ of the needle trajectory should be minimized.	$\beta_{i}\to min$	
S2	The needle trajectory length $L_{i}$ should be minimized.	$L_{i}\to min$	
S3	The insertion angle $\theta_{i}$ should be as close to 90° as possible.	$\theta_{i}\to 90^{\circ }$	
S4	The needle trajectory should maximize the distance to critical structures.	Distance $\left(p_{i},S_{critica}\right)$→max	
S5	In multi‐needle ablation planning, pull‐back trajectories should be prioritized when trajectories overlap (with the same insertion skin entry point and angles but different lengths).	$\left\{\begin{array}{c} f\left(p_{pullback_{i,j}}\right)\;for\;i\neq j\;and\;O\;\left(p_{i,\;}p_{j}\right)=1\\ f(p_{i},\;p_{j}) \end{array}\right.$ $f\left(p_{pullback_{i,j}}\right)>f\left(p_{i},p_{j}\right)$	the prioritization score of each needle trajectory $f(p_{i},\;p_{j},p_{pullback})$ determining the priority order; the overlap of needle trajectory $p_{i}$ and $p_{j}$ represented by $O\;(p_{i,\;}p_{j})=1$

#### 3.3.2 Multi‐objective optimization of needle trajectories

The set of candidate needle trajectories derived from the initial planning is further optimized based on five soft clinical constraints (Figure 4b), including the vertical deflection and insertion angles, trajectory length, safe distance to critical structures, and pull‐back technique (Table 1). Among these constraints, the pull‐back technique is the top priority in multi‐needle trajectory planning when the planned trajectory paths overlap. The overlap of needle trajectory is defined as the trajectory paths with the same insertion skin entry point and angles but different lengths, as shown in Figure 3b. Since patient individual differences lead to soft constraints with distinguishing priorities, our method allows doctors to set their preferred weights for soft constraints in trajectory optimization before planning. By adjusting these weights, doctors can interactively improve the optimization process to prioritize certain objectives over others for patients with unique clinical considerations, such as a tumor adjacent to critical structures or with specific anatomical challenges, making it closer to a global optimum for the specific patient, rather than settling for a general compromise.

A two‐stage needle trajectory optimization process is developed for higher practicability in clinical settings. In the first stage, the Non‐dominated Sorting Genetic Algorithm II (NSGA‐II)^36^ is performed to generate a set of Pareto‐optimal solutions satisfying all the soft constraints on different levels, detailed in Materials S2. Afterward, these Pareto‐optimal solutions can be optionally selected by the clinician's preference or our second‐stage automatic optimization. In the second stage, the Pareto‐optimal solutions derived from first‐stage optimization are ranked according to different weighted constraints using the TOPSIS method. In the TOPSIS‐based optimization, the final optimal solution is determined by the relative closeness in the weighted decision matrix: larger relative closeness represents a higher probability of the optimal solution. The steps of the TOPSIS method are described in Materials S3.

## 4 EVALUATION

To evaluate our planning method, preoperative CT data including 30 tumors from 23 patients were retrospectively selected in our institution. As described in Section 2.1, the tumor and anatomical structures are segmented and annotated from CT images in advance. The tumor size ranging from 7.8 to 35.5 mm met the size range applicable to the proposed multi‐needle planning. The fixed ablation ellipsoid with the long‐ and short‐axis radius of 40  and 30 mm, respectively, was set according to the clinician's experience in our ablation planning. The following parameters were used in our evaluation: the safety margin of 5 mm, the maximum length of the ablation needle $L_{max}$ 140 mm, the liver capsule insertion angle $\theta_{i}$ ranging 20°–90°, horizontal deflection angle $\alpha_{i}$ ranging 30°–150°, vertical deflection angle $\beta_{i}$ ranging 0°–45°, transhepatic trajectory depth $d_{i}$ > 5 mm, the minimum distance between needles $d_{ij}\;5\;\mathrm{mm}.$ In the trajectory optimization, the pull‐back technique (S5) was prioritized, followed by the vertical deflection angle, trajectory length, liver capsule insertion angle, and distance to critical structures constraints (S1‐4) with equal weights.

### 4.1 Evaluation metrics

**FIGURE 5:**
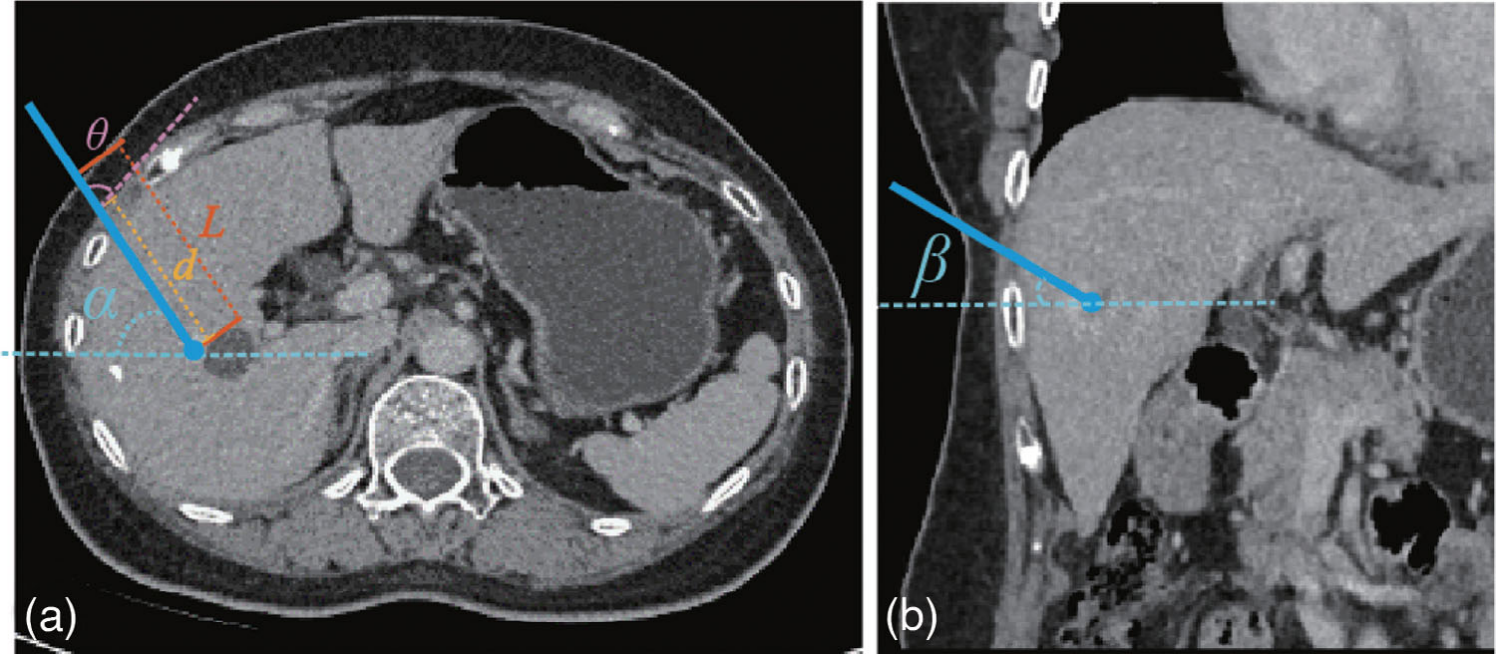
Illustration of the constraint parameters, including the needle trajectory length *L*, the transhepatic trajectory depth *d*, the liver capsule insertion angle *θ*, the horizontal deflection angle *α* (a), and the vertical deflection angle *β* (b).

The ablation number, coverage percentage (CP), ablation efficiency (AE), and ablation boundary distance (ABD) are analyzed for each single‐ and multi‐ablation plan to evaluate the effectiveness, efficiency, and safety of ablation planning. All the generated needle trajectories are quantitatively assessed using the trajectory number, trajectory length (TL), minimum distance to critical structures (DTC), and insertion angles (vertical deflection and liver capsule insertion angles) (Figure 5). Additionally, the planning time for each tumor is recorded. The evaluation metrics are listed below:

(1) Ablation number
(2) Trajectory number: the number of ablation and trajectory are unequal when implementing the pull‐back technique.
(3) Trajectory length (TL): the straight‐line distance from the skin entry point to the target point.
(4) Minimum distance to critical structures (DTC): the shortest vertical distance between the 3D volume vertices of the critical structures and the needle trajectory.
(5) Vertical deflection and liver capsule insertion angle: the angle between the needle trajectory and the axial CT slice, and the tangent line of the liver capsule within the axial CT slice, respectively.
(6) Coverage percentage (CP): the percentage of the ablated volume within the treatment zone to the treatment zone.
(7) Ablation efficiency (AE): the ratio of the ablated volume within the treatment zone to the total ablated zone.
(8) Ablation boundary distance (ABD): the distance between the boundary of the ablation zone and the treatment zone.

Additionally, the clinical acceptability of the planning results was verified by two experienced interventional radiologists (Radiologist 1, Dr Z.B., and Radiologist 2, Dr X.L., with more than 8 and 15 years of experience in percutaneous hepatic ablation, respectively). The two radiologists independently rated the results at 0, 1, and 2 satisfaction scores representing clinically unacceptable, acceptable, and preferred plans, respectively.

### 4.2 Clinical validation

Additionally, our proposed planning system was applied to five consecutive patients with focal HCC lesions undergoing CT‐guided percutaneous liver tumor microwave ablation (MWA) in January 2024 in our institution to further validate its clinical feasibility and practicability. This prospective study was approved by the Institutional Review Committee of our institution (2021‐BJYYEC‐367‐04) and written informed consent was acquired from all patients after a thorough explanation of the potential risks and benefits before procedures. Preoperative multiphase contrast‐enhanced CT images were acquired and preprocessed 30 min before procedures. With a safety margin of 5 mm, our planning method generated the ablation and trajectory plans as the reference of the operator (Dr X.L.), who performed all the procedures. As the 18‐gauge MWA antenna (Vision Medical Devices R&D Center, Nanjing, China) was used, the fixed ablation ellipsoid zone was set to 40 mm×30 mm×30 mm. All planning results were checked and accepted by the operator. With the assistance of a 3D visualization tool, the doctor inserted the ablation needle according to planned needle trajectories and accomplished the ablation with the power ranging 30–50 W and the duration ranging 4–6 min. After the MWA was terminated by the operator, abdominal CT scans were performed immediately, 24 , and 48 h after procedures to radiologically evaluate tumor coverage and complications. All patients were followed up with abdominal enhanced CT examinations at 3 and 6 months after procedures until July 2024 to monitor the local tumor recurrence.

### 4.3 Implementation

Our planning method was written in Python 3.7 on a desktop PC (Intel(R) Xeon(R), 2.10 GHz, 128G RAM, Windows 10 64bit). All the results were obtained from the desktop PC equipped with a Gold 6230R 2.10 GHz CPU with 128 GB RAM and a GeForce RTX‐3090 GPU with 24GB memory. The visualization of the planning results was presented using the Visualization Toolkit (VTK).^37^

## 5 RESULTS

As shown in Table 2, the dataset involved 23 patients with 30 HCC tumors, including 13 male and 10 female patients, with a median age of 56 years (range 31–68). There were 17 patients (73.9%) with a single tumor and 6 (26.1%) with multiple lesions. An equal number of 11 cases (47.8%) were clinically diagnosed with BCLC stage 0 and A, respectively. Seventeen (73.9%) and 5 (21.7%) patients were diagnosed with CNLC Ia and Ib stage. Only one patient (Patient 6) classified as BCLC B and CNLC IIa underwent ablation treatment by the recommendation of CNLC guidelines.^38^ According to clinical consensus, liver ablation treatment is feasibly applied to tumors with a diameter ≤ 5 cm, thus the tumor size was 18.2 ± 7.5 mm (range 7.8–35.5), meeting applicable the size range. The tumors were located at I‐VIII hepatic segments (according to Couninaud's segmentation^39^).

**TABLE 2:** Clinical characteristics of the patients and lesions.

Characteristics	
Patients (Lesions)	23 (30)
Age (years)^a^	56 (31–68)
Gender	
Male	13 (56.5%)
Female	10 (43.5%)
Number of lesions	
Single lesion	17 (73.9%)
Multiple lesions	6 (26.1%)
BCLC stage	
0	11 (47.8%)
A	11 (47.8%)
B	1 (4.3%)
CNLC stage	
Ia	17 (73.9%)
Ib	5 (21.7%)
IIa	1 (4.3%)
Tumor size, (mm)^b^	18.2 ± 7.5 (7.8–35.5)

Unless otherwise indicated, data are expressed as number of patients, followed by the percentages in parentheses.

Abbreviations: BCLC, Barcelona Clinic Liver Cancer; CNLC, China Liver Cancer Staging.

^a^ Data are expressed as median with the range in parentheses.

^b^ Data are expressed as mean ± standard deviation with the range in parentheses.

**FIGURE 6:**
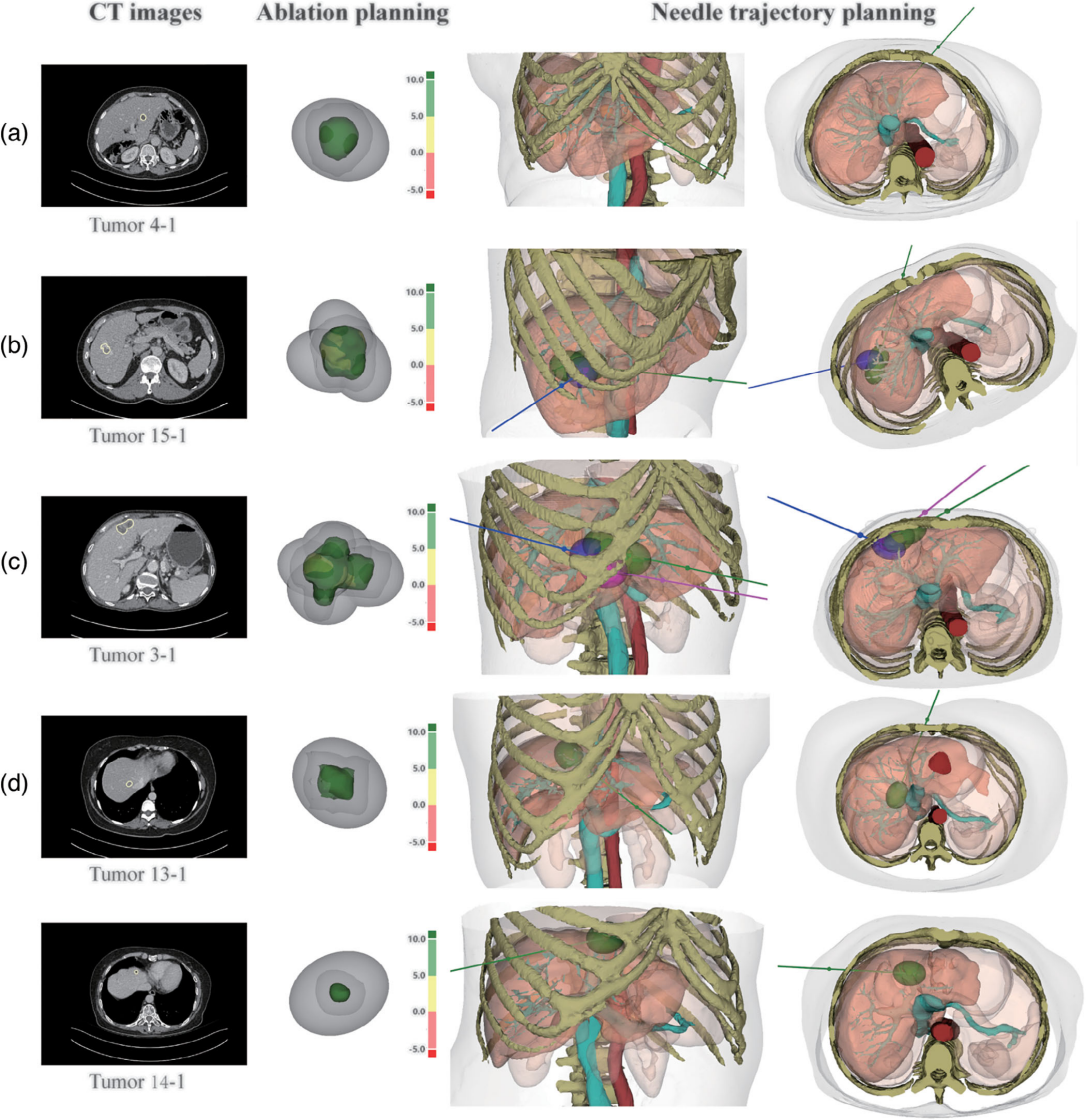
Visualization of the ablation and needle trajectory planning results. (a) The single‐needle planning results (Tumor 4‐1). (b) The multi‐needle planning results include a pull‐back trajectory represented by a green line (Tumor 15‐1). (c) The planning results for the largest tumor (Tumor 3‐1, with a tumor size of 35.5 mm) in our Test Dataset, where the pull‐back trajectory is represented by a green line. (d) The planning results for the tumor located at the hepatic dome (Tumor 13‐1 & 14‐1). Each column demonstrates the axial CT images including the target tumor contoured by the white circle (first column), the ablation planning results (second column) where the color distance map shows the distance between the tumor and ablation zones, and the needle trajectory planning results in 3D views (third and fourth columns).

Our proposed method generated 18 single‐needle and 12 multi‐needle ablation and trajectory planning results for the 30 tumors. As shown in Table 3, tumor size, TV, and TZV of the target lesions with single‐needle and multi‐needle trajectory planning were 13.3 ± 3.5  and 25.5 ± 5.8 mm, 1081.2 ± 810.1 and 5586.9 ± 2947.4 mm^3^, and 6287.2 ± 2676.3 and 17813.9 ± 6381.2 mm^3^, respectively. Among 12 multi‐needle plans, the number of ablation zones ranged from 2 to 4, whereas the trajectory number varied from 1 to 3 due to 4 pull‐back trajectory paths derived from our method. The planning results are representatively illustrated in Figure 6.

**TABLE 3:** Summary of the trajectory planning results and evaluation.

	All trajectory planning	Single‐needle trajectory planning	Multi‐needle trajectory planning
Number	30	18	12
Tumor size (mm)	18.2 ± 7.5 (7.8–35.5)	13.3 ± 3.5 (7.8–19.1)	25.5 ± 5.8 (20.0–35.5)
Tumor volume (mm^3^)	2883.5 ± 2953.0 (220.9–10 056.3)	1081.2 ± 810.1 (220.9–2983.2)	5586.9 ± 2947.4 (2630.8–10 056.3)
Treatment zone volume (mm^3^)	10897.9 ± 7254.7 (3024.0–28 223.3)	6287.2 ± 2676.3 (3024.0–12 123.5)	17813.9 ± 6381.2 (10 691.5–28 223.3)
Trajectory length (mm)	83.6 ± 30.3 (35.0–138.6)	86.4 ± 31.1 (35.2–138.6)	82.0 ± 30.2 (35.0–123.9)
Minimum distance to critical structures (mm)	16.7 ± 8.8 (5.0–35.2)	14.4 ± 6.2 (5.0–28.5)	18.0 ± 9.9 (5.0–35.2)
Vertical deflection angle (°)	4.5 ± 6.1 (0.0–19.8)	9.0 ± 7.2 (0.1–19.8)	1.4 ± 2.1 (0.0–9.1)
Liver capsule angle (°)	85.3 ± 3.7 (73.1–89.9)	85.8 ± 3.8 (73.1–89.3)	84.9 ± 3.6 (73.9–89.9)
Coverage percentage (%)	99.8 ± 0.3 (99.1–100.0)	100	99.6 ± 0.4 (99.1–100.0)
Ablation efficiency (%)	40.5 ± 14.2 (15.0–65.4)	33.3 ± 14.1 (15.0–63.5)	48.8 ± 8.2 (38.4–65.4)
First quantile of ABD (mm)	5.7 ± 2.3 (1.2–10.2)	7.3 ± 1.9 (4.5–10.2)	4.3 ± 1.5 (1.2–6.2)
Mean value of ABD (mm)	9.3 ± 1.5 (6.9–11.9)	9.7 ± 1.4 (7.2–11.9)	8.7 ± 1.4 (6.9–11.7)
Third quantile of ABD (mm)	13.2 ± 0.9 (10.3–14.8)	13.1 ± 1.0 (10.3–14.1)	13.4 ± 0.6 (12.5–14.8)
Time (s)	23.6 ± 20.6 (4.2–112.9)	16.6 ± 7.8 (4.2–33.9)	34.1 ± 28.8 (7.0–112.9)

Data are expressed as mean ± standard deviation with the range in parentheses.

Abbreviation: ABD , ablation boundary distance.

In the ablation planning, all the simulated ablation zones achieved complete tumor coverage (100% and 99.6% coverage for TZV in single‐needle and multi‐needle ablation planning, respectively) without involving the critical structures. Our ablation planning achieved an average AE of 40.5%, with a lower AE of 33.3 ± 14.1% for single‐needle ablations compared with 48.8 ± 8.2% efficiency in multi‐needle ablation planning. The first, mean, and third quantiles of ABD were 5.7, 9.3, and 13.2 mm, respectively (7.3/9.7/13.1 and 4.3/8.7/13.4 mm for single‐needle and multi‐needle ablation planning, respectively).

In needle trajectory planning, no collisions with critical structures or between trajectories occurred. The TL and DTC of generated trajectory paths were 83.6 ± 30.3 mm (range 35.0–138.6) and 16.7 ± 8.8 mm (5.0–35.2), respectively (86.4 ± 31.1  and 14.4 ± 6.2 mm for single‐needle trajectories, and 82.0 ± 30.2  and 18.0 ± 9.9 mm for multi‐needle trajectories). As for insertion angles, our method attained an average vertical deflection angle of 4.5 ± 6.1° (range 0.0–19.8) and an average liver capsule angle of 85.3 ± 3.7° (range 73.1–89.9).

The average planning time for each tumor was 23.6 ± 20.6 s (16.6 ± 7.8 and 34.1 ± 28.8 s for single‐needle and multi‐needle plans, respectively). The summary of planning results and detailed information on each single‐needle and multi‐needle ablation and trajectory plan are quantitatively listed in Table 3, and Tables 4 and 5, respectively.

**TABLE 4:** Detailed information on single‐needle trajectory planning results and evaluation.

Patient and tumor characteristics						Planning										Satisfaction score	
										Insertion Angles							
Patient	Tumor case	Location	Tumor maximum diameter (mm)	TV (mm^3^)	TZV (mm^3^)	Ablation number	Trajectory number	Trajectory length (mm)	DTC (mm)	Vertical deflection Angle (°)	Liver capsule Angle (°)	Coverage percentage (%)	Ablation efficiency (%)	First quantile/ mean/ third quantile of ABD (mm)	Time (s)	R1	R2
1	1	III	17.0	1822.0	8830.2	1	1	65.7	28.5	1.2	88.8	100	46	[4.5/8.2/12.4]	17.6	2	2
1	2	II	11.8	805.0	5525.5	1	1	66.6	6.9	3.3	82.1	100	28.2	[7.7/10.0/13.1]	10.5	2	2
2	1	IV	11.3	676.5	5530.4	1	1	35.2	13.9	5.1	84.2	100	29.3	[8.7/10.3/13.7]	11.7	1	1
4	1	IV	16.6	1755.0	8493.2	1	1	77.7	5.7	1.3	89.3	100	44.7	[5.6/8.2/13.2]	4.8	2	2
5	1	IV	10.4	354.1	3447.4	1	1	36.1	20.9	0.7	88.3	100	20.6	[9.0/11.1/13.8]	12.2	2	2
5	2	VI	9.7	289.1	3352.7	1	1	81.2	17.1	0.1	86.3	100	15.3	[9.4/11.3/14.1]	22.6	2	2
6	3	VI	18.2	1180.4	6802.8	1	1	66.6	13.6	6.6	87.9	100	36.3	[4.6/9.2/12.9]	12.6	2	2
7	1	IV	18.4	2730.2	11095.5	1	1	68.2	9.4	5.6	87.2	100	58.8	[5.1/7.4/11.0]	28.1	2	2
10	1	II	10.7	499.3	4082.0	1	1	57.5	13.4	11.3	87.9	100	20.3	[8.8/10.8/13.6]	13	1	1
11	1	IV	15.9	1446.4	8335.8	1	1	100.6	21.7	16	86.4	100	43.2	[6.5/8.8/12.9]	8	1	2
12	1	IV	11	576.5	4712.9	1	1	94.7	16.6	15.1	73.1	100	26.8	[7.8/10.4/13.9]	16.5	2	1
13	1	VIII dome	13.5	1032.3	5897.8	1	1	121.7	11.4	14.7	88.2	100	31.9	[6.9/9.6/12.6]	19.2	2	2
14	1	IV dome	7.8	220.9	3050.7	1	1	74.4	5	18.9	82.4	100	15.7	[10.2/11.9/13.8]	4.2	2	2
19	1	II	9	260.8	3024.0	1	1	113.4	21	18.1	85.9	100	15	[10.1/11.6/14.1]	20.3	2	2
21	1	V	19.1	2983.2	12123.5	1	1	138.6	12.7	7	87.2	100	63.5	[4.9/7.2/10.3]	33.9	2	2
21	2	VI	13.2	867.1	5951.7	1	1	112.0	8.5	19.8	84.5	100	33.2	[6.1/9.5/13.7]	18	2	1
22	1	VIII	14.1	1300.9	7497.6	1	1	136.6	16.6	16.1	88.9	100	38.5	[6.8/9.0/12.6]	22.9	1	1
23	1	VI	12.4	662.5	5415.7	1	1	108.1	16.3	0.7	85.3	100	31.6	[8.0/10.4/13.7]	22.6	2	2

Abbreviations: ABD, ablation boundary distance; AE, ablation efficiency; CP, coverage percentage; DTC, minimum distance to critical structures; R1 , Radiologist 1; R2 , Radiologist 2; TL, trajectory length; TMD, tumor maximum diameter; TV, tumor volume TZV, treatment zone volume;.

**TABLE 5:** Detailed information on multi‐needle trajectory planning results and evaluation (including pull‐back trajectory planning).

Patient and tumor characteristics						Planning											Satisfaction score	
											Insertion angles							
Patient	Tumor case	Location	Tumor maximum diameter (mm)	TV (mm^3^)	TZV (mm^3^)	Ablation number	Trajectory number	Path no.	Trajectory length (mm)	DTC (mm)	Vertical deflection Angle (°)	Liver capsule angle (°)	Coverage percentage (%)	Ablation efficiency (%)	First quantile/mean/third quantile of ABD (mm)	Time (s)	R1	R2
3	1	IV, V	35.5	9047.6	25962.5	4	3	P1^a^	52.9/56.3	29.5/29.8	0.0	84.1	99.8	59.3	[4.9/9.9/13.0]	112.9	1	1
								P2	41.5	35.1	1.8	82.2						
								P3	39.1	25.6	3.6	78.1						
3	2	VI	21.0	3528.5	14339.7	2	2	P1	117.1	13.5	0.1	82.4	99.2	48.8	[4.3/8.1/13.6]	25.3	2	2
								P2	35.0	11.0	0.2	85.9						
6	1	I	23.2	4684.2	15988.6	2	2	P1	119.0	10.0	0.3	87.5	99.4	45.2	[2.3/8.4/13.4]	19.6	2	1
								P2	108.9	14.6	1.5	86.3						
6	2	V	20.1	2864.2	11639.9	2	2	P1	75.7	5.0	0.4	87.9	100	40.2	[6.2/9.1/13.8]	19.1	2	2
								P2	90.6	13.1	0.0	73.9						
8	1	II, III	20.0	3130.4	12721.7	2	2	P1	55.3	6.2	2.0	84.4	100	43.9	[5.5/9.0/13.1]	11.1	2	2
								P2	77.3	11.8	0.3	84.4						
8	2	IV	20.7	2812.2	11427.4	2	1	P1^a^	50.1/51.9	8.1/8.1	9.1	83.3	99.1	54.3	[4.2/7.3/12.5]	7.0	2	2
9	1	VI	32.8	9840.4	28223.3	3	3	P1	58.4	30.6	0.1	84.8	99.1	65.4	[1.2/6.9/13.4]	32.9	2	2
								P2	66.6	14.9	0.3	83.6						
								P3	101.9	22.4	0.3	83.2						
15	1	V	29.5	6806.2	19516.2	3	2	P1^a^	56.4/57.2	29.1/29.1	1.8	88.5	99.7	56.3	[2.3/6.9/13.0]	59.1	2	2
								P2	53.5	27.0	0.5	88.0						
16	1	VII	21	3702.4	14627.2	2	2	P1	119.0	10.0	0.3	87.5	99.4	45.2	[2.3/8.4/13.4]	19.6	2	2
								P2	97.5	11.2	6.6	87.8						
17	1	VIII	29.3	10056.3	25837.3	3	3	P1	112.5	21.9	0.2	85.0	100	43.8	[5.4/10.4/13.4]	32.4	1	1
								P2	71.0	11.8	0.7	81.3						
								P3	123.9	9.8	2.1	83.5						
18	1	IV	31.5	7939.9	22791.2	3	2	P1^a^	122.2/116.1	35.2/33.1	0.3	89.0	99.6	44.9	[5.1/11.7/14.8]	46.7	2	2
								P2	101.3	17.3	0.5	89.9						
20	1	VIII	20.8	2630.8	10691.5	2	2	P1	109.5	7.4	2.6	86.7	100	38.4	[5.7/8.8/13.6]	23.6	2	1
								P2	121.7	7.9	1.3	89.1						

Abbreviations: ABD, ablation boundary distance; AE, ablation efficiency; CP, coverage percentage; DTC, minimum distance to critical structures; R1 , Radiologist 1; R2 , Radiologist 2; TL, trajectory length; TMD, tumor maximum diameter; TV, tumor volume TZV, treatment zone volume;

^a^ Path represents the “pull‐back” trajectory planning.

Additionally, all the planning results were rated as clinically acceptable by two experienced radiologists, with all satisfaction scores ≥ 1. Notably, 20 plans (66.7%) scored 2 by both radiologists were considered clinically preferred, supporting the clinical practicability of our method. There was an excellent agreement between the radiologists (Cohen's Kappa 0.636, 95% CI: 0.326–0.946, *p*<0.001).

**FIGURE 7:**
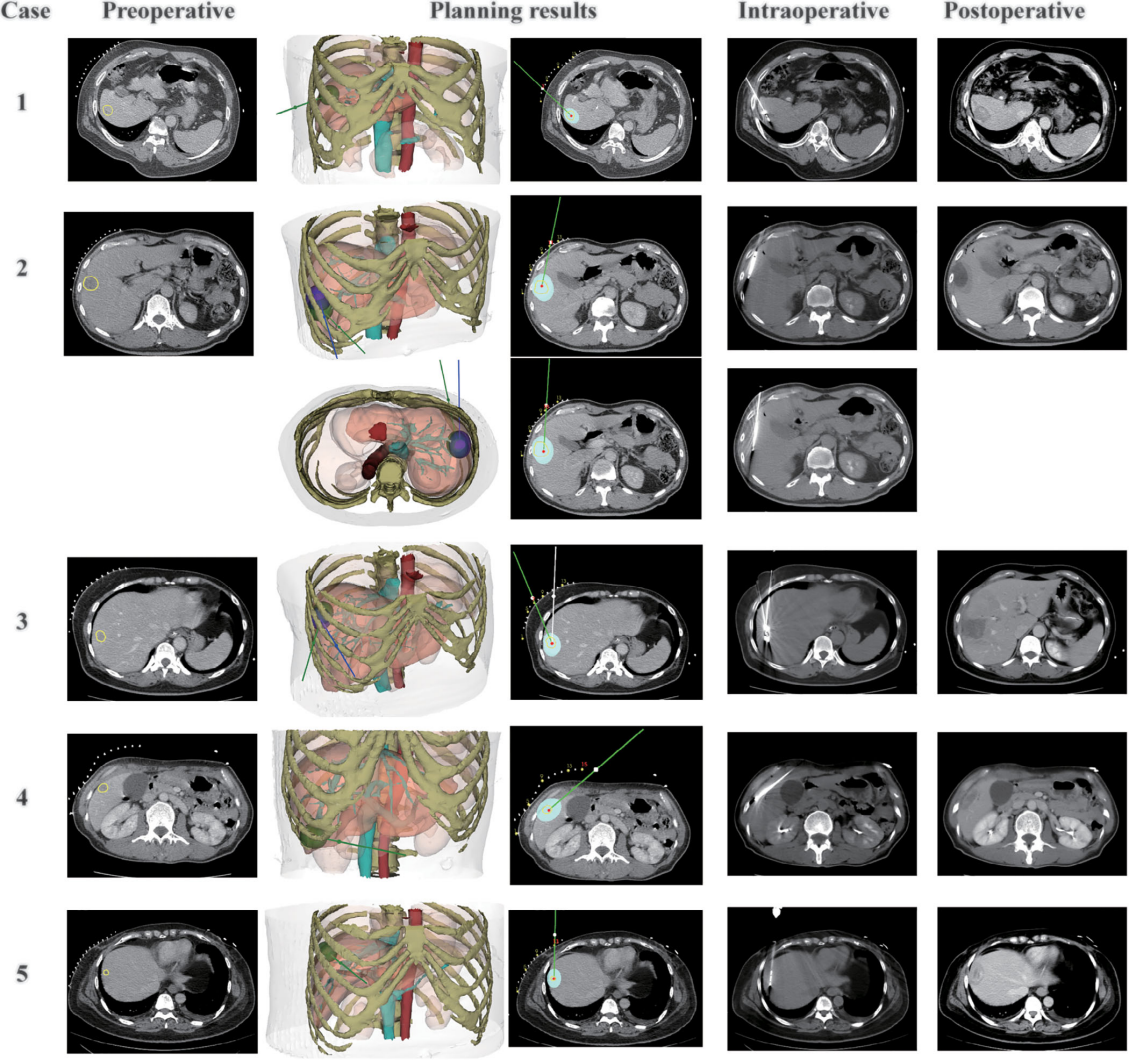
The planning results of clinical cases. Preoperative CT images demonstrate the target tumor segmented by the yellow line. The planning results include 3D visualization and 2D projection on the axial CT images of the generated plans. The intraoperative and postoperative images show the actual needle trajectory and ablation regions.

In clinical validation, our planning method was performed successfully with complete tumor coverage on CT images, and no intraoperative and postoperative complications occurred in five clinical cases. The performance of the planning method was recognized by the doctor. The planning results are shown in Table 6 and Figure 7. Additionally, no local tumor recurrence was observed during follow‐up.

**TABLE 6:** The planning results for clinical cases.

Case	Tumor size (mm)	Tumor volume (mm^3^)	Treatment zone volume (mm^3^)	Ablation number	Trajectory number	Path no.	Trajectory length (mm)	DTC (mm)	Vertical deflection angle (°)	Liver capsule angle (°)	Coverage percentage (%)	Ablation efficiency (%)	First quantile/ mean/ third quantile of ABD (mm)	Time (s)	Complications
1	18.3	1556.0	7338.1	1	1	P1	87.4	16.3	0.1	84.0	100	51.4	2.5/4.7/8.9	26.9	No
**2**	34.9	18633.5	27458.7	2	2	P1	83.0	21.9	2.8	83.4	100	39.3	3.4/7.5/11.4	64.5	No
						P2	62.2	19.3	3.2	83.8					
**3**	23.8	4176.6	14253.7	2	2	P1	87.0	15.2	3.7	84.1	99.8	50.3	3.3/4.5/11.4	52.3	No
						P2	91.3	15.9	6.4	83.9					
**4**	19.9	1501.8	6891.4	1	1	P1	85.0	23.2	0.5	82.8	100	38.8	2.4/6.2/12.0	27.4	No
**5**	13.7	864.8	4807.6	1	1	P1	68.5	9.5	0.9	83.1	100	32.8	2.4/4.9/12.1	23.1	No

Abbreviation: DTC, minimum distance to critical structures.

## 6 DISCUSSION

We proposed a full‐automatic ablation and trajectory planning method that integrated the desired tumor‐enclosing ablation zone planning and multiple clinical constraints to generate clinically feasible plans for hepatic tumor ablation within a short time. Based on the augmented Lagrangian and iterative method, the generated ablation plans fulfilled the complete tumor coverage and critical structure avoidance and reached a trade‐off between the ablation number and damage to surrounding healthy tissues. The needle trajectory results, derived from the initial planning and two‐stage MOO, comprehensively satisfied hard constraints and considered different‐weighted soft constraints to obtain the final optimal solution.

Through the initial planning, the feasible skin entry points of candidate needle trajectories are restricted by six hard constraints, including critical structure collision, needle length, insertion angles, transhepatic depth, puncture scope, and needle collision constraints, greatly improving the computational speed and iterative efficiency; then, the Pareto‐TOPSIS MOO comprehensively considers the vertical deflection and insertion angles, trajectory length, distance to critical structures, and pull‐back technique, to sequentially obtain a set of Pareto‐optimal solutions and the final optimal plan. Notably, the TOPSIS‐based approach is innovatively introduced to determine the preferable solution satisfying multiple clinical objectives and priorities from Pareto‐optimal solutions, offering a potential solution to MOO problems.

The planning time of our method meets the clinical requirements of preoperative planning. Regardless of all single‐needle results within 35 s, less than 1 min was consumed for more complicated multi‐needle planning for larger tumors (except Tumor 3‐1, with four ablations and three trajectories planning for 112.9 s), thereby making it clinically possible to obtain planning results within a short time. As the ablation and trajectory number should be minimized to reduce complication risks and mitigate healthy tissue damage, more than four ablations or punctures are not clinically recommended.^38^ Considering the pull‐back technique, our method effectively reduced the ablation and trajectory numbers with a maximum of 4 and 3 for larger tumors, respectively, limiting surgical complications and costs. Additionally, sequential analysis of ablation and trajectory numbers procedurally simplifies computational complexity and accelerates the target point determination for each trajectory.

In ablation zone planning, simulated ablation zones without overlapping critical structures satisfied the safe ablation requirements, with a 99.8% treatment zone coverage and a 40.5% AE for all the tumors. Under the complete coverage constraint, our single‐ and multi‐ablation planning reached 100% and 99.6% coverage of the TZV, respectively, ensuring the effectiveness of tumor ablation. Since the optimized configuration of multiple ablation zones was comprehensively considered for larger tumors to balance the ablation number and AE, the AE in multi‐ablation plans was 15% higher compared to single‐ablation results, mainly due to the fixed ablation range in simulation. Our algorithm technically supports adjustable geometric parameters of the ablation zone depending on the clinician's preference, contributing to higher flexibility for surgical planning. Furthermore, the mean ABDs<10 mm indicate the reliability of less damage to healthy tissues.

Multiple clinical constraints comprehensively considering the safety, feasibility, and flexibility, are introduced into our trajectory planning, including the feasible skin region determination and Pareto‐TOPSIS trajectory optimization. All the generated trajectories did not interfere with critical organs or large blood vessels, and other trajectories if any. The TL, DTC, vertical deflection, and liver capsule angles for each trajectory were quantitatively evaluated. Particularly, the TL and two insertion angles varied within a clinically reasonable range, enabling the feasibility and safety of our method; the adequate DTC allowed for higher fault tolerance and flexibility in surgical planning, improving the safety of needle insertion. Of note, for the tumors (Tumor 13‐1 and 14‐1, Figure 6d) located at the hepatic dome that is notoriously harder to approach, the generated trajectories with larger vertical deflection angles were considered the clinically preferable choice by both radiologists. According to the radiologists’ evaluation, our method can generate feasible and preferable plans for tumors with various sizes, shapes, locations, and surrounding structures, verifying the generalizability and applicability to clinical scenarios.

The validation results from clinical cases showed complete tumor coverage without complications and no local tumor recurrence during follow‐up, strengthening the clinical practicability of our planning system. As a full‐automatic planning system, our method can not only generate preoperative plans rapidly but also integrate with navigation or robotic surgical systems to facilitate surgical procedures.

The performance comparison to the recent planning methods^26^, ^27^, ^29^, ^31^ is briefly demonstrated in Table 7. Our planning method can generate the Pareto‐TOPSIS final optimal solution compared to relatively sub‐optimal solutions obtained from other approaches. Regardless of variations in the tumors and implementing environments in previous studies, our proposed full‐automatic method integrated more comprehensive clinical constraints and pull‐back techniques, reduced the computing time to less than 30 s, minimized the ablation and trajectory numbers under complete tumor coverage, and was evaluated with the largest dataset and clinical cases. Due to the fixed ablation zone parameters, our method achieved a slightly lower but acceptable AE, but reached the trade‐off between the AE and ablation number, resulting in more practical ablation plans.

**TABLE 7:** Performance comparison to recent planning methods.

Method	Application scope	Planning time (s)	Automatic Level	Clinical constraints	Pull‐back technique	Optimized results	Validation Data (patient/tumor)	Ablation number	Trajectory number	Trajectory length (mm)	Tumor coverage (%)	Average ablation efficiency (%)
Chen et al.^26^	Small and large tumors	41	Semi‐automatic	H1,H2,H5	No	The targets and the corresponding conical puncture regions. Not the global optimal solution	18/18	2.9 (1–6) for 10 cases	2.9 (1–6) for 10 cases	NM	100	67.8
Liang et al.^27^	Small and large tumors	About 480	Full‐automatic	H1,H2,H3,H6	Yes	Probably a sub‐optimal solution due to the optimization time limit. Not the global optimal solution	9/20	1.3 (1–3) for Plan A; 3.6 (2–7) for Plan B	1.1 (1–2) for Plan A; 2.2 (1–3) for Plan B	Less than 150 mm	100	39.3 for Plan A; 53.7 for Plan B
Li et al.^28^	Large tumors	About 300–3180	Full‐automatic	H1,H2,H3,H6	No	Multiple solutions sometimes. Not the global optimal solution	5/6	2.3 (1–4)	2.3 (1–4)	61.4	100	NA
Li et al.^31^	Small and large tumors	41.4	Full‐automatic	H1‐6	Yes	Pareto optimal solutions. Not the global optimal solution	7/10	4.0 (2–6)	3.1 (2–5)	69.5	100	60.2
Li et al.^29^	Small and large tumors	59	Full‐automatic	H1	Yes	A clinically feasible solution. Not the global optimal solution	15/18	1.4 (1–3)	1.3 (1–3)	NM	100	33.2
Our method both	Small and large tumors	23.6	Full‐automatic	H1‐6	Yes	Pareto‐TOPSIS optimal solution	23/30	1.3 (1–4)	1.5 (1–3)	83.6	100	40.5 (33.3 for single‐needle; 48.8 for multi‐needle)

Data are expressed as numbers with the range in parentheses.

Abbreviation: NM , not mentioned in the study.

Our proposed method has several limitations. First, our multi‐ablation planning simulated the configuration of multiple ablation zones that perform simultaneously, without considering interactions between ablation needles or the deformation of tumors and surrounding structures caused by the sequential ablations. Second, the target point of each needle trajectory in our method is set as the centroid of each ablation ellipsoid from a single ablation zone or configuration of multiple ablation zones. However, the ablation needle tip generally passes through the tumor with an uncertain needle length outside the tumor edge in practice, leading to no accurate modeling for the actual target point. Third, since our performance evaluation was retrospectively performed on single‐center data, extensive validation on larger or multicenter datasets is warranted to further confirm the safety and effectiveness.

## 7 CONCLUSION

The proposed multi‐stage automatic ablation and needle trajectory planning algorithm for hepatic tumor ablation can generate a final optimal plan satisfying multiple clinical constraints within a short time, potentially facilitating clinician preoperative planning.

## CONFLICT OF INTEREST STATEMENT

The authors have no relevant financial or non‐financial interests to disclose.

The authors have completed the STROBE reporting checklist.

## Supporting information



Supporting Information

Supporting Information

Supporting Information

## Data Availability

Patient data currently cannot be publicly accessible due to privacy protection. Reasonable requests for the datasets and materials used in this study can be addressed to the corresponding author.
