# Supplementary material for: Multi‐stage automatic and rapid ablation and needle trajectory planning method for CT‐guided percutaneous liver tumor ablation
Source: Med Phys. 2024 Oct 10;52(1):113–30. doi: 10.1002/mp.17450 (PMC11700007; doi:10.1002/mp.17450)
Supplement: Supplementary file 1 — Supporting Information [file MP-52-113-s001.docx]

**Steps of the iterative solution**

The augmented Lagrangian function is as follows:

$$\mathcal{L}_{\mathcal{c}}\left( \{p_{i},\theta_{i}, \phi_{i}, \psi_{i}{\}}_{i=1}^{n}, \boldsymbol{\lambda},\mu\right) =R\left( \{p_{i},\theta_{i},\phi_{i},\psi_{i}{\}}_{i=1}^{n} \right)-\lambda_{1}\text{G(}\mathbf{E}\text{) +}\lambda_{2}H(\mathbf{E})-\frac{\mu}{2}\left( {\text{G(}\mathbf{E}\text{) }}^{2}+{H\left( \mathbf{E} \right)}^{2} \right)$$

Where $\lambda=\{\lambda_{1}, \lambda_{2}\}$ denotes the Lagrange multiplier and $\mu$ denotes the penalty parameter used to adjust the weight of penalty term**.**

The iterative solution steps are as follows:

Initialize the ellipsoid parameters $\left\{ p_{i},\theta_{i},\phi_{i},\psi_{i} \right\}_{i=1}^{n}$ to be solved.

1. Combining random generation and heuristic strategies based on medical prior knowledge, the centroid position of each ellipsoid is initially uniformly distributed within the tumor, and the ellipsoid angles are initialized around the tumor's long axis following a Gaussian distribution.
2. Optimize the subproblem.

For fixed $\lambda^{k}$ and $\mu$, the BFGS (Broyden--Fletcher--Goldfarb--Shanno) algorithm is utilized to solve the unconstrained optimization subproblem. The BFGS algorithm is a widely used quasi-Newton method, which is an efficient optimization algorithm that uses second-order derivative information to accelerate convergence without explicitly calculating the Hessian matrix. In each iteration, the centroid position and angle of each ablation ellipsoid are determined, thus the objective function gradually approaches the optimal solution satisfying the tumor coverage rate and other constraints.

1. Update the Lagrange multiplier.

$$\lambda_{1}^{k+1} = \lambda_{1}^{k} + \mu{\text{G(}E\text{)}}^{k+1}$$

$$\lambda_{2}^{k+1} = \lambda_{2}^{k} + \mu{H\text{(}E\text{)}}^{k+1}$$

1. Check the convergence condition.

If $\text{G(}E\text{)}$ and $H\left( E \right)$ is sufficiently small, terminate the iteration process; otherwise, increase the penalty parameter 𝑐 and return to step 2.
