# Supplementary material for: Multi‐stage automatic and rapid ablation and needle trajectory planning method for CT‐guided percutaneous liver tumor ablation
Source: Med Phys. 2024 Oct 10;52(1):113–30. doi: 10.1002/mp.17450 (PMC11700007; doi:10.1002/mp.17450)
Supplement: Supplementary file 2 — Supporting Information [file MP-52-113-s003.docx]

**The Non-dominated Sorting Genetic Algorithm II (NSGA-II)** is a popular multi-objective optimization algorithm widely used in fields requiring optimization under multiple competing objectives, such as in medical planning and engineering design. The key strength of NSGA-II lies in its capability to identify a set of Pareto-optimal solutions rather than a single solution, allowing decision-makers to select from a range of trade-off solutions. The NSGA-II works as follows:

1. **Initial Population Generation**:

NSGA-II begins with creating an initial population $P_{0}$ of size $N$, where each individual $x_{i}\in P_{0}$represents a potential solution. Each individual is encoded as a vector $\mathbf{x}_{\mathbf{i}}=\left[ x_{i1},x_{i2},\ldots,x_{id} \right]$, where $d$ is the number of decision variables (e.g., insertion angles, trajectory lengths, etc.).

1. **Evaluation of Objectives:**

Each solution $\mathbf{x}_{\mathbf{i}}$ is evaluated based on multiple objective functions $f_{j}\left( \mathbf{x}_{\mathbf{i}} \right)$, where$j=1,2,\ldots,m$, $m$ corresponds to different soft constraints in the needle trajectory planning. The objective functions might include:

$$f_{1}\left( x_{i} \right)=\text{minimize the distance to critical structures}$$

$$f_{2}\left( x_{i} \right)=\text{minimize the trajectory length}$$

and so on.

1. **Non-dominated Sorting:**

The solutions are sorted into different Pareto fronts based on dominance. A solution $x_{i}$ dominates another solution $x_{j}$(denoted as $x_{i}\prec\mathbf{x}_{\mathbf{j}}$) if:

$$f_{k}\left( x_{i} \right)\leq f_{k}\left( x_{j} \right)\quad\forall k\in\{1,2,\ldots,m\}$$

and

$$f_{l}\left( x_{i} \right)<f_{l}\left( x_{j} \right)\quad\text{for at least one }l\in\{1,2,\ldots,m\}$$

Solutions that are not dominated by any other solutions form the first Pareto front, representing the best trade-offs in the current population. The next set of non-dominated solutions forms the second Pareto front, and so on.

1. **Crowding Distance Calculation:**

Within each Pareto front, NSGA-II calculates a crowding distance for each solution, which measures how close each solution is to its neighbors in the objective space. Solutions with a larger crowding distance are preferred to maintain diversity in the population.

1. **Selection, Crossover, and Mutation:**

NSGA-II selects solutions based on their rank (Pareto front) and crowding distance for reproduction. Selected solutions undergo crossover (combining two parent solutions) and mutation (small random changes) to produce a new generation of solutions, helping explore the solution space and avoid premature convergence to suboptimal solutions.

1. **Iterative Process:**

The algorithm iteratively refines the population over multiple generations. With each iteration, the population evolves, leading to a more refined set of Pareto-optimal solutions that better satisfy the multiple objectives.

NSGA-II's ability to efficiently explore and exploit the solution space while maintaining diversity makes it an ideal choice for multi-objective optimization problems like needle trajectory planning. It provides a robust set of solutions, enabling clinicians to balance competing objectives and choose the most appropriate plan for their patients.
