# Supplementary material for: Multi‐stage automatic and rapid ablation and needle trajectory planning method for CT‐guided percutaneous liver tumor ablation
Source: Med Phys. 2024 Oct 10;52(1):113–30. doi: 10.1002/mp.17450 (PMC11700007; doi:10.1002/mp.17450)
Supplement: Supplementary file 3 — Supporting Information [file MP-52-113-s002.docx]

**Steps of the TOPSIS method**

The steps of the TOPSIS method are shown as follows:

1. **Determination of soft-constraint weights**

The first step is determining the weights for each soft constraint condition S1-S5 (listed in Table 1) based on requirements and priorities in the specific clinical situation.

1. **Development of decision matrix**

A decision matrix $X=\left[ x_{ij} \right]$ is developed in the multi-objective analysis, where each row represents a Pareto-optimal solution and each column represents the score of a soft constraint condition, with $x_{ij}$ denoting the score of the solution $i$ on soft constraint condition $j$ .

1. **Normalization of decision matrix**

To eliminate the influence of dimension, the decision matrix is normalized using the formula below:

$r_{ij}=\frac{x_{ij}}{\sqrt{\sum_{i=1}^{m} x_{ij}^{2}}}$

where $m$ denotes the number of Pareto-optimal solutions.

1. **Weighted normalization of decision matrix**

The normalized matrix $R=\left[ r_{\mathrm{ij}} \right]$ is then multiplied by the weight vector $W=\left[ w_{\mathrm{ij}} \right]$ to obtain the weighted normalized matrix $V=\left[ v_{\mathrm{ij}} \right]$, where $v_{\mathrm{ij}} = w_{j}\cdot r_{\mathrm{ij}}$.

1. **Determination of the ideal and anti-ideal solutions**

The ideal solution:

$$A^{+}=v_{1}^{+},v_{2}^{+},\ldots v_{n}^{+}$$

The anti-ideal solution:

$$A^{-}=v_{1}^{-},v_{2}^{-},\ldots v_{n}^{-}$$

The ideal and anti-ideal solutions are determined by identifying each soft constraint condition's maximum and minimum values, respectively.

1. **Euclidean distances of each solution to the ideal and anti-ideal solutions**

The Euclidean distances of each solution to the ideal solution $d_{i}^{+}$ and the anti-ideal solution $d_{i}^{-}$ are calculated using the formulas below, respectively:

$$d_{i}^{+}=\sqrt{\sum_{j=1}^{n} \left( v_{\mathrm{ij}}-v_{j}^{+} \right)^{2}}$$

$$d_{i}^{-}=\sqrt{\sum_{j=1}^{n} \left( v_{\mathrm{ij}}-v_{j}^{-} \right)^{2}}$$

1. **Computation of the relative closeness**

The relative closeness $C_{i}^{+}$for each solution is computed using the formula:

$$C_{i}^{+} = \frac{d_{i}^{-}}{d_{i}^{+}+d_{i}^{-}}$$

where $d_{i}^{+}$ and $d_{i}^{-}$ denote the ideal and anti-ideal solution, respectively. The larger the relative closeness $C_{i}^{+}$ , the better the solution $i$.

1. **Optimal solution selection**

Finally, all the Pareto-optimal solutions are ranked according to their relative closeness $C_{i}^{+}$, and the solution with the highest relative closeness is selected as the optimal solution.
